# Analysis of disease characteristics of a large patient cohort with congenital generalized lipodystrophy from the Middle East and North Africa

**DOI:** 10.1186/s13023-024-03084-2

**Published:** 2024-03-13

**Authors:** Saif Al Yaarubi, Afaf Alsagheir, Azza Al Shidhani, Somaya Alzelaye, Nadia Alghazir, Imad Brema, Hussain Alsaffar, Mohammed Al Dubayee, Awad Alshahrani, Yasmine Abdelmeguid, Omneya M. Omar, Najya Attia, Elham Al Amiri, Jamal Al Jubeh, Albandari Algethami, Haya Alkhayyat, Azad Haleem, Mouza Al Yahyaei, Ines Khochtali, Saleha Babli, Ahmed Nugud, Nandu Thalange, Sarah Albalushi, Nadia Hergli, Asma Deeb, Majid Alfadhel

**Affiliations:** 1 Oman Medical Specialty Board, Muscat, Oman; 2 Pediatrics Department, King Faisal Specialist Hospital and Research Centre, Riyadh, Saudi Arabia; 3 Department of Child Health, Division of Endocrinology, Sultan Qaboos University Hospital, Al-Khod, Muscat, Oman; 4 Center of Endocrinology and Diabetes Mellitus, Al-Qunfudah General Hospital, Makkah Province, Al-Qunfudah, Saudi Arabia; 5 Department of Pediatrics, Faculty of Medicine, Tripoli University Hospital, University of Tripoli, Tripoli, Libya; 6 Obesity, Endocrine, and Metabolism Center, King Fahad Medical City, Riyadh, Saudi Arabia; 7 College of Medicine, King Saud Bin Abdulaziz University for Health Sciences, Riyadh, Saudi Arabia; 8 King Abdullah International Medical Research Center, Riyadh, Saudi Arabia; 9 Department of Medicine, Ministry of the National Guard-Health Affairs, Riyadh, Saudi Arabia; 10 Faculty of Medicine, Alexandria University, Alexandria, Egypt; 11 King Abdullah International Medical Research Center, Jeddah, Saudi Arabia; 12 Al Qassimi Women & Children Hospital, Sharjah, United Arab Emirates; 13 Sheikh Khalifa Medical City, Abu Dhabi, United Arab Emirates; 14 King Fahad Military Hospital, Jeddah, Saudi Arabia; 15 Bahrain Defence Force Royal Medical Services, Riffa, Bahrain; 16 University of Duhok/College of Medicine, Duhok, Iraq; 17 National Diabetes and Endocrine Center, Royal Hospital, Muscat, Oman; 18 Internal Medicine and Endocrinology Department, Fattouma Bourguiba University Hospital, University of Monastir, Monastir, Tunisia, Monastir, Tunisia; 19 Prince Sultan Military Medical City, Riyadh, Saudi Arabia; 20 Al Jalila Children’s Specialty Hospital, Dubai, United Arab Emirates; 21 Department of Medicine, Mohammed Bin Rashid University, Dubai, United Arab Emirates; 22 Amryt Pharmaceuticals DAC, Dublin, Ireland; 23 Pediatric Endocrine Division, Sheikh Shakhbout Medical City & College of Medicine and Health Sciences, Khalifa University, Abu Dhabi, United Arab Emirates; 24 Genetic and Precision Medicine Department, King Abdullah Specialized Children Hospital, King Abdulaziz Medical City, Ministry of National Guard Health Affairs (MNGHA), Riyadh, Saudi Arabia; 25 College of Medicine, King Abdullah International Medical Research Center (KAIMRC), King Saud bin Abdulaziz University for Health Sciences, Ministry of National Guard Health Affairs (MNGHA), Riyadh, Saudi Arabia; 26 King Saud Bin Abdulaziz University for Health Sciences, Ministry of National Guard Health Affairs (MNGH), Riyadh, Saudi Arabia

**Keywords:** Congenital generalized lipodystrophy, HbA1c, Generalized lipodystrophy, Metabolic disease, Organ system abnormalities, Middle East, North Africa, Triglycerides

## Abstract

**Background:**

Congenital generalized lipodystrophy (CGL) is a rare inherited disease characterized by a near-total absence of adipose tissue and is associated with organ system abnormalities and severe metabolic complications. Here, we have analyzed the disease characteristics of the largest CGL cohort from the Middle East and North Africa (MENA) who have not received lipodystrophy-specific treatment.

**Methods:**

CGL was diagnosed clinically by treating physicians through physical assessment and supported by genetic analysis, fat loss patterns, family history, and the presence of parental consanguinity. Data were obtained at the time of patient diagnosis and during leptin-replacement naïve follow-up visits as permitted by available medical records.

**Results:**

Data from 43 patients with CGL (37 females, 86%) were collected from centers located in eight countries. The mean (median, range) age at diagnosis was 5.1 (1.0, at birth–37) years. Genetic analysis of the overall cohort showed that CGL1 (n = 14, 33%) and CGL2 (n = 18, 42%) were the predominant CGL subtypes followed by CGL4 (n = 10, 23%); a genetic diagnosis was unavailable for one patient (2%). There was a high prevalence of parental consanguinity (93%) and family history (67%) of lipodystrophy, with 64% (n = 25/39) and 51% (n = 20/39) of patients presenting with acromegaloid features and acanthosis nigricans, respectively. Eighty-one percent (n = 35/43) of patients had at least one organ abnormality; the most frequently affected organs were the liver (70%, n = 30/43), the cardiovascular system (37%, n = 16/43) and the spleen (33%, n = 14/43). Thirteen out of 28 (46%) patients had HbA1c > 5.7% and 20/33 (61%) had triglyceride levels > 2.26 mmol/L (200 mg/dl). Generally, patients diagnosed in adolescence or later had a greater severity of metabolic disease versus those diagnosed during childhood; however, metabolic and organ system abnormalities were observed in a subset of patients diagnosed before or at 1 year of age.

**Conclusions:**

This analysis suggests that in addition to the early onset of fat loss, family history and high consanguinity enable the identification of young patients with CGL in the MENA region. In patients with CGL who have not received lipodystrophy-specific treatment, severe metabolic disease and organ abnormalities can develop by late childhood and worsen with age.

**Supplementary Information:**

The online version contains supplementary material available at 10.1186/s13023-024-03084-2.

## Introduction

Lipodystrophy syndromes comprise a rare group of diseases defined by the loss of adipose tissue. These diseases are traditionally classified according to the extent of fat loss (generalized, affecting the whole body, or partial, affecting selected areas) and etiology (genetic or acquired) [1, 2]. Recent assessment of international electronic medical record databases estimated the global prevalence of generalized lipodystrophy at 0.23 cases/million. In the same study, analysis of literature searches yielded a prevalence of 0.96 cases/million for generalized lipodystrophy in the European population [3]. Congenital generalized lipodystrophy (CGL) is an autosomal recessive disease in which the onset of fat loss typically occurs at birth or shortly thereafter [1, 2]. The estimated global prevalence of CGL is 1 case per 10 million, although prevalence may be higher in geographical regions (including parts of the Middle East and South America) where parental consanguinity is more frequent [4–7].

The lack of functioning adipose tissue in CGL causes the ectopic storage of fats resulting in severe metabolic complications (e.g., insulin resistance, diabetes mellitus and hypertriglyceridemia), and organ system abnormalities (e.g., non-alcoholic fatty liver disease [NAFLD], acute pancreatitis, and renal and cardiovascular disease) which contribute to morbidity and mortality [4, 8, 9]. Deficiency in leptin (a key hormone regulator of satiety, and glucose and lipid homeostasis, produced by adipocytes) can cause hyperphagia and exacerbate symptoms [1, 10–12].

CGL is classically divided into four subtypes based on genetic etiology. CGL type 1 (CGL1) is associated with variants in the 1-acylglycerol-3-phosphate O-acyltransferase 2 (*AGPAT2*) gene which encodes an enzyme involved in triglyceride synthesis [1, 13, 14]. CGL type 2 (CGL2) is associated with variants in the Berardinelli-Seip congenital lipodystrophy 2 (*BSCL2*) gene, which encodes seipin, a protein with a role in lipid droplet formation and adipocyte differentiation [1, 13, 14]. Variants in the caveolin 1 (*CAV1*) gene and the caveolae-associated protein 1 gene (*CAVIN1,* previously called the polymerase I and transcript release factor [*PTRF*] gene) give rise to CGL types 3 and 4 (CGL3, CGL4), respectively, with the products of both genes involved in intracellular trafficking and lipid metabolism [1, 13, 14]. CGL1 and CGL2 are the most common subtypes of CGL, with CGL3 and CGL4 reported only in a small number of patients [1, 13].

In addition to the near-total loss of body fat, the four CGL subtypes share several characteristics that classically define the CGL phenotype. These included prominent veins and muscles, hepatomegaly, splenomegaly, acanthosis nigricans, acromegaloid appearance, and eruptive xanthomas [13]. Clinical features that can help distinguish CGL subtypes include: (1) a loss of metabolically active fat with sparing of mechanically functioning fat in CGL1; (2) more severe metabolic disease in CGL2; (3) preservation of mechanical and bone marrow fat, short stature, vitamin D resistance, hypocalcemia, hypomagnesemia and achalasia in CGL3; and (4) myopathy, skeletal and gastrointestinal abnormalities, cardiac arrhythmias and elevated creatine kinase levels in CGL4 with milder metabolic disease in some cases versus other subtypes [1, 13, 15, 16].

The treatment of CGL focuses on the amelioration or prevention of metabolic and organ system abnormalities. Management includes a combination of lifestyle modification (e.g., diet and exercise) and pharmacotherapy (e.g., insulin and other anti-diabetic medications, lipid-lowering and cardiovascular therapies) [1, 8, 12]. Recombinant human methionyl leptin (metreleptin) is the only specific therapy approved as an adjunct to diet as replacement therapy for the metabolic complications of lipodystrophy [1, 8, 12]; however, the availability of this therapy is limited in some regions. Consequently, assessment of the disease characteristics of patients with CGL who have not received lipodystrophy-specific treatments, including leptin-replacement therapy, provides an opportunity to further evaluate the onset of comorbidities and the burden of disease.

The natural history of generalized lipodystrophy has been examined in large patient cohorts, primarily from the Americas, Europe, and Asia with limited data on patients from across the Middle East and North Africa (MENA) region [2, 11, 17–26]. To address this paucity of data, we have examined the physical, clinical, and metabolic characteristics of the largest lipodystrophy-specific treatment-naïve CGL cohort from the MENA region reported to date. We present our findings in the context of earlier observational studies describing the disease characteristics of CGL in comparable patient populations.

## Methods

### Ethics statement

Approval for participation in this retrospective research was obtained from the ethics committee or institutional review board at participating medical centers. The Declaration of Helsinki and Guideline for Good Clinical Practice from the International Conference on Harmonization were adhered to throughout this project [27, 28]. As per local regulations, written consent was not necessary for the use of retrospective anonymized (unidentifiable) data; however, all subjects (or their legal guardians) provided written consent for genetic testing.

### Research objectives and cohort

This was a retrospective, observational, noninterventional, multicenter medical record analysis designed to evaluate the disease characteristics of patients with CGL from the MENA region. Data were obtained from the medical charts of patients treated at participating medical centers in eight countries (Bahrain, Egypt, Iraq, Libya, Oman, Saudi Arabia, Tunisia, and the United Arab Emirates [UAE]).

CGL was diagnosed clinically by treating physicians. The principal clinical diagnostic criterion for CGL was generalized loss of fat. Other physical characteristics used to support a diagnosis of CGL included pronounced muscular appearance, acromegaloid features, acanthosis nigricans, and hirsutism. Family history and the presence of parental consanguinity also aided diagnosis.

### Genetic analysis

Genetic analyses were conducted at accredited laboratories using proprietary in-house methods and analytical pipelines or were performed at participating medical centers using previously published methods (Additional file 1: Information) [17, 29, 30].

### Variables collected and analyzed

Data were obtained at the time of patient diagnosis and during leptin-replacement naïve follow-up visits as permitted by available medical records. Patients were not receiving lipodystrophy-specific treatment (including leptin-replacement therapy) at the time of data collection. Patient demographic data included sex, country of origin, age at diagnosis, year of diagnosis, and medication history. The physical characteristics associated with CGL assessed were acromegaloid features, acanthosis nigricans and the presence of hirsutism. Organ systems abnormalities were evaluated for the cardiovascular system (e.g., left ventricular hypertrophy, patent foramen ovale, atrial septal defects), the liver (e.g., NAFLD, hepatomegaly), the spleen (e.g., splenomegaly), the pancreas (e.g., pancreatitis), the renal system (e.g., albuminuria, nephromegaly, proteinuria), bones and/or joints (e.g., dysplasia, metaphysis, osteoporosis) and the reproductive system (e.g., polycystic ovaries, irregular menses).

The metabolic parameters analyzed were glycated hemoglobin (HbA1c), triglycerides, total cholesterol, alanine aminotransferase (ALT), aspartate aminotransferase (AST), gamma-glutamyl transferase (GGT), fasting plasma glucose (FPG), fasting insulin and creatine kinase levels. Hematological parameters comprised hemoglobin, white blood cell count, platelet count, serum calcium and serum creatinine levels. All metabolic and hematological parameters were measured in laboratories at each participating medical center using standardized methods with appropriate quality control procedures.

Data on background medication use was recorded for each patient, where available. These data included information on anti-diabetic medication use (e.g., insulin aspart or insulin glargine, metformin, pioglitazone, or dapagliflozin), lipid-lowering medication and treatment (e.g., gemfibrozil, statins, omega 3 fatty acids, fenofibrate, low-fat formula), cardiovascular medication (e.g., angiotensin-converting enzyme [ACE] inhibitors, beta-blockers, angiotensin receptor blockers) and anti-coagulants (e.g., warfarin).

### Statistical analysis

Statistical analyses were conducted using Stata version 15.1 (Stata Corp LLC, Texas, USA) and the statistical package contained in Microsoft Excel. All available data were anonymized prior to statistical analysis. Mean ± standard deviation (SD), and median (range, minimum–maximum) values were used to summarize continuous variables (i.e., patient demographics, metabolic laboratory data). Categorical variables summarized the number and proportion of patients with physical features and organ system abnormalities associated with lipodystrophy, and background medication use. Assessment of the proportion of patients with elevated metabolic parameters was based on previously published thresholds: ALT (> 35 IU/L and > 55 IU/L), AST (> 35 IU/L and > 48 IU/L), HbA1c (> 5.7%, and > 6.5%), triglycerides (> 1.69 mmol/L [> 150 mg/dL], > 2.26 mmol/L [> 200 mg/dL] and > 5.65 mmol/L [> 500 mg/dL]) and total cholesterol > 5.18 mmol/L (> 200 mg/dl) [11, 22, 31, 32].

Data were summarized for the whole cohort, CGL subtypes, and by the age of diagnosis (i.e., ≤ 1, < 12 and ≥ 12 years of age). Statistical comparisons between groups evaluated differences in disease presentation. Due to the small sample sizes, categorical variables were compared between groups using Fisher’s exact test. Continuous variables had positively skewed distributions and were analyzed using the Kruskal–Wallis test. If a significant overall difference between groups was observed, *post-hoc* comparisons between pairs of groups were subsequently made using either Fisher’s exact tests (categorical variables) or Mann–Whitney tests (continuous variables). To account for multiple comparisons for each outcome, Bonferroni adjustment was made to pairwise *P*-values comparisons. A *P*-value of less than 0.05 was accepted as statistically significant.

## Results

### Patient demographics of the overall MENA CGL cohort

In total, data from 43 (37 females, 86%) patients with CGL were collected from medical centers located in eight MENA countries. Patient demographics for the overall CGL cohort are shown in Table 1. Thirty-eight patients (88%) originated from the Middle East (Bahrain, Iraq, Oman, Palestine, Saudi Arabia, and the UAE; the single patient from Palestine was treated at a medical center in the UAE); the remaining five patients (12%) were from North Africa (Egypt, Libya, and Tunisia). Saudi Arabia (n = 20; 47%) and Oman (n = 13, 30%) were the countries with the highest patient representation. The proportion of patients with consanguineous parents was 93% (n = 40/43) and 67% (n = 29/43) of patients had a recorded family history of lipodystrophy.

**Table 1:** Patient characteristics of the overall CGL cohort (*N* = 43)

Characteristic	Overall cohort
*Age at CGL diagnosis, years*	
n_(available)_	43
Mean (± SD)	5.1 (± 8.9)
Median (range)	1.0 (at birth–37)
*Sex, n/n*_*(available)*_ *(%)*	
Female	37/43 (86)
Male	6/43 (14)
*Country/State, n/n*_*(available)*_ *(%)*	
Egypt	1/43 (2)
Iraq	1/43 (2)
Kingdom of Bahrain	1/43 (2)
Libya	3/43 (7)
Oman	13/43 (30)
Palestine*	1/43 (2)
Saudi Arabia	20/43 (47)
Tunisia	1/43 (2)
United Arab Emirates (UAE)	2/43 (5)
Parental consanguinity, n/n_(available)_ (%)	40/43 (93)
Family history of lipodystrophy, n/n_(available)_ (%)	29/43 (67)
*Height* (cm)	
n_(available)_	32
Mean (± SD)	92 (± 40)
Median (range)	79 (45–174)
*Weight (kg)*	
n_(available)_	33
Mean (± SD)	18.2 (± 19.9)
Median (range)	9.5 (2.4–68.0)
*Body mass index* (kg/m^2^)	
n_(available)_	30
Mean (± SD)	16.5 (± 3.7)
Median (range)	16.6 (9.9–25.0)
CGL subtype**	
CGL1 (*AGPAT2*)	14/42 (33)
CGL2 (*BSCL2*)	18/42 (43)
CGL4 (*CAVIN1*)	10/42 (24)
*Physical features, n/n*_*(available)*_* (%)*	
Acromegaloid features	25/39 (64)
Acanthosis nigricans	20/39 (51)
Hirsutism^†^	7/37 (19)
*Organ system abnormalities, n/n*_*(available)*_* (%)*	
At least one abnormality	35/43 (81)
Bones and/or joints	8/43 (19)
Cardiovascular system	16/43 (37)
Liver	30/43 (70)
Hepatomegaly	27/43 (63)
Pancreas	4/43 (9)
Renal system	10/43 (23)
Reproductive system^‡^	7/9 (78)
Spleen	14/43 (33)
Splenomegaly	14/43 (33)
*Medication history, n/n*_*(available)*_ *(%)*	
Anticoagulant	1/43 (2)
Antidiabetic medication	9/43 (21)
Cardiovascular medication	7/43 (16)
Lipid-lowering medication/treatment	10/43 (23)

^*^This patient was treated in a medical center in the UAE

^**^A genetic diagnosis was unavailable for the single patient from Tunisia

^†^Female patients only

^‡^Data presented only for post-pubertal males (aged > 12 years) and females (aged > 11 years)

Data were obtained at the time of patient diagnosis and during leptin-replacement naïve follow-up visits as permitted by available medical records. Data are presented as n (%) unless otherwise indicated; percentages based on the number of patients for whom data on each characteristic were available. *AGPAT2*, 1-acylglycerol-3-phosphate O-acyltransferase 2; *BSCL2*, seipin; *CAVIN1*, caveolae associated protein 1; *CGL* Congenital generalized lipodystrophy, *n* Number of patients exhibiting the disease characteristic, *n*_(available)_, number of patients with available data

The mean (± SD) and median (range) ages at diagnosis for the overall cohort were 5.1 (± 8.9) and 1.0 (at birth–37) years, respectively. Over half of the patients (51%, n = 22/43) were diagnosed ≤ 1 year old and 81% (n = 35/43) of patients were diagnosed before the age of 12 years. Eight patients (19%) were diagnosed aged ≥ 12 years, of whom five were diagnosed between 12 and 18 years of age, with one patient each diagnosed at 21, 36 and 37 years of age. The median height (79 cm; range, 45–174 cm), weight (9.5 kg; range, 2.4–68.0 kg) and body mass index (16.6 kg/m^2^, range 9.9–25.0 kg/m^2^) of the overall cohort reflect the young age at which patients were diagnosed.

Classification of CGL subtypes based on genotype (and supported by clinical presentation and family history by the treating physician) was performed for 42 patients (Table 1). CGL2 (n = 18, 43%) was the predominant subtype, followed by CGL1 (n = 14, 33%) and CGL4 (n = 10, 24%). At the time of analysis, genetic variants in the *AGPAT2*, *BSCL2*, *CAV1* or *CAVIN1* genes were not detected in the single case from Tunisia (who had a well-established history of CGL) and hence a CGL subtype was not assigned to this patient. Genetic variants recorded for the MENA cohort are shown in Additional file 1: Table 1. The identities and genomic locations of lipodystrophy-associated DNA sequence variants were documented for 38 of the 42 patients (90%) based on available medical records; of these, 36 patients had variants that were classified as pathogenic while two patients (one each with CGL1 and CGL4) had variants of uncertain significance. The available medical records for the remaining four patients recorded the name of the affected gene only (i.e., *AGPAT2*, *BSCL2*, or *CAVIN1*).

### Assessment of physical characteristics, organ abnormalities and metabolic parameters in the overall MENA CGL cohort

Patients in the overall cohort exhibited a range of physical characteristics associated with generalized lipodystrophy such as acromegaloid features (64%, n = 25/39), acanthosis nigricans (51%, n = 20/39), and hirsutism (19%; n = 7/37; all females diagnosed with CGL at or before the age of 2 years). Most patients (81%, n = 35/43) had at least one organ system abnormality. The liver was the most frequently affected organ (70%, n = 30/43), followed by the cardiovascular system (37%, n = 16/43), the spleen (33%, n = 14/43), the renal system (23%, n = 10/43), bones and/or joints (19%, n = 8/43), and the pancreas (9%, n = 4/43). Of the nine post-pubertal patients (i.e., female patients > 11 years and male patients > 12 years) available for analysis, seven (78%, all female) had reproductive system abnormalities (Table 1).

Assessment of background medication showed that 23% of patients (n = 10/43) were receiving lipid-lowering treatments, with 21% (n = 9/43) and 16% (n = 7/43) receiving anti-diabetic and cardiovascular medications, respectively. Only one patient (2%) was taking anticoagulants at the time of data collection while a single patient with CGL4 had received zoledronic acid for the treatment of bone complications (Table 1).

Metabolic and hematological data were available for a subset of patients in the overall cohort (Table 2 and Additional file 1: Table 2). Mean HbA1c (7.3 ± 3.1%), FPG (8.9 ± 5.5 mmol/L), triglycerides (6.1 ± 7.3 mmol/L), AST (53 ± 32 IU/L) and ALT (66 ± 56 IU/L) values were elevated. HbA1c levels > 5.7% and > 6.5% were observed in 46% (n = 13/28) and 39% (n = 11/28) of patients, while 70% (n = 23/33) and 61% (n = 20/33) had triglycerides > 1.69 mmol/L and > 2.26 mmol/L, respectively. Total cholesterol > 5.18 mmol/L occurred in 31% (n = 9/29) of patients. Thirty-seven percent (n = 13/35) of patients had ALT > 55 IU/L and 47% (n = 16/34) had AST > 48 IU/L. Values for hematological parameters (i.e., hemoglobin, white blood cells, platelets, serum calcium and serum creatinine) were within the normal range.

**Table 2:** Hematological and metabolic parameters for the overall CGL cohort

	Overall CGL cohort		
Parameter	n_(available)_	Mean (± SD)	Median (range)
Hemoglobin (g/dL)	36	12.3 (± 2.0)	12.2 (8.2–16.6)
Platelet count (10^9^ platelets/L)	36	361 (± 155)	330 (79–739)
Serum calcium (mmol/L)	29	2.4 (± 0.4)	2.4 (0.6–2.8)
Serum creatinine (µmol/L)	32	33 (± 17)	30 (12–80)
White blood cell count (10^9^ cells/L)	36	9.2 (± 4.0)	8.6 (4.1–19.7)
ALT (IU/L)	35	66 (± 56)	46 (14–232)
AST (IU/L)	34	53 (± 32)	46 (15–187)
Creatine kinase (IU/L)	20	981 (± 1514)	150 (16–6000)
Fasting insulin (pmol/L)	17	384 (± 511)	79 (13–1611)
FPG (mmol/L)	23	8.9 (± 5.5)	5.4 (4.0–19.3)
GGT (IU/L)	16	66 (± 44)	49 (13–175)
HbA1c (%)	28	7.3 (± 3.1)	5.7 (4.5–14.3)
Total cholesterol (mmol/L)	29	4.9 (± 2.1)	4.3 (2.1–13.2)
Triglycerides (mmol/L)	33	6.1 (± 7.3)	2.9 (0.9–31.0)

Data were obtained at the time of patient diagnosis and during leptin-replacement naïve follow-up visits as permitted by available medical records. Reference ranges: Fasting insulin, < 111 pmol/L (< 16 mUI/ml); FPG, 3.9–5.5 mmol/L (70–99 mg/dl); HbA1c, < 5.7%; Triglycerides, < 1.69 mmol/L (< 150 mg/dl); Total cholesterol, < 5.18 mmol/L (< 200 mg/dl) [11, 22, 31, 32]. *ALT* Alanine aminotransferase, *AST* Aspartate aminotransferase, *CGL* Congenital generalized lipodystrophy, *FPG* Fasting plasma glucose, *GGT* Gamma-glutamyl transferase, *HbA1c* Glycated hemoglobin, *n* Number of patients exhibiting the disease characteristic, *n*_(available)_, Number of patients with available data, *SD* Standard deviation

### Comparison of disease characteristics and metabolic parameters between CGL groups

Table 3 presents a comparison of the disease characteristics and metabolic parameters between the three CGL subtypes detected in the MENA cohort (demographic and anthropometric data are given in Additional file 1: Table 3). Patients with CGL1 were, on average, diagnosed later than those with CGL2 and CGL4 (CGL1, mean age: 9.7 ± 10.6 years; CGL2, mean age: 3.3 ± 8.4 years; CGL4, mean age: 0.8 ± 0.8 years). The proportion of patients with acromegaloid features did not differ between the three CGL subtypes (*P* = 0.91); however, acanthosis nigricans was reported in patients with CGL1 (64%, n = 9/14) and CGL2 (71%, n = 10/14) but was not reported in any patients with CGL4 (*P* = 0.001). Differences in the prevalence of cardiovascular abnormalities were observed between subtypes (*P* = 0.001), with a higher prevalence in CGL4 (60%, n = 6/10) and CGL2 (56%, n = 10/18) versus CGL1 (0%, n = 0/14). Analysis of background medication showed that more patients with CGL1 were receiving anti-diabetic medication compared with the other CGL subtypes (CGL1: 43%, n = 6/14; CGL2: 11%, n = 2/18; CGL4: 0%, n = 0/10; *P* = 0.02), with no differences observed between CGL subtypes for the other background medications assessed (*P* > 0.05).

**Table 3:** Comparison of disease characteristics and metabolic parameters by CGL subtype

	CGL1 subtype	CGL2 subtype	CGL4 subtype	*P*-value
*Age at diagnosis, years*				
n_(available)_	14	18	10	
Mean (± SD)	9.7 (± 10.6)	3.3 (± 8.4)	0.8 (± 0.8)	
Median (range)	7 (0.1–37)	0.8 (0.1–36)	0.5 (at birth–2)	
*Sex, n/n(available)* *(%)*				
n_(available)_	14	18	10	
Female	13 (93%)	16 (89%)	7 (70%)	
Male	1 (7%)	2 (11%)	3 (30%)	
*Physical features, n/n*_*(available)*_ *(%)*				
Acanthosis nigricans	9/14 (64)	10/14 (71)	0/10 (0)	0.001
Acromegaloid features	9/14 (64)	10/14 (71)	6/10 (60)	0.91
*Organ system abnormalities, n/n*_*(available)*_ *(%)*				
Bones/joints	4/14 (29)	2/18 (11)	2/10 (20)	0.48
Cardiovascular system	0/14 (0)	10/18 (56)	6/10 (60)	0.001
Liver	11/14 (79)	13/18 (72)	5/10 (50)	0.39
Hepatomegaly	8/14 (57)	13/18 (72)	5/10 (50)	0.51
Pancreas	2/14 (14)	1/18 (6)	0/10 (0)	0.59
Renal system	5/14 (36)	5/18 (28)	0/10 (0)	0.09
Spleen	3/14 (21)	6/18 (33)	5/10 (50)	0.34
Splenomegaly	3/14 (21)	6/18 (33)	5/10 (50)	0.34
*Medication history, n/n*_*(available)*_ *(%)*				
Anticoagulants	0/14 (0)	1/18 (6)	0/10 (0)	1.00
Anti-diabetic medication	6/14 (43)	2/18 (11)	0/10 (0)	0.02
Cardiovascular medication	1/14 (7)	4/18 (22)	2/10 (20)	0.57
Lipid-lowering medication/treatment	6/14 (43)	3/18 (17)	1/10 (10)	0.16
*Metabolic parameters*				
FPG (mmol/L)				
n_(available)_	12	9	1	0.62*
Mean (± SD)	9.7 (± 6.3)	7.4 (± 3.8)	4.0 (± 0.0)	
Median (range)	6.1 (4.2–19.3)	5.4 (4.0–14.0)	4.0 (4.0–4.0)	
HbA1c (%)				
n_(available)_	11	12	4	0.41
Mean (SD)	8.2 (± 3.8)	6.9 (± 2.6)	5.3 (± 0.4)	
Median (range)	5.8 (4.6–14.3)	5.8 (4.5–12.0)	5.3 (4.8–5.7)	
> 5.7%, n/n_(available)_ (%)	6/11 (55)	6/12 (50)	0/4 (0)	0.19
> 6.5%, n/n_(available)_ (%)	5/11 (45)	5/12 (42)	0/4 (0)	0.37
> 8.0%, n/n_(available)_ (%)	5/11 (45)	3/12 (25)	0/4 (0)	0.23
Total cholesterol (mmol/L)^*^				
n_(available)_	12	13	3	0.04
Mean (± SD)	5.7 (± 2.7)	4.5 (± 1.2)	3.2 (± 0.9)	
Median (range)	5.2 (3.8–13.2)	4.3 (3.0–7.7)	3.6 (2.1–3.8)	
Triglycerides (mmol/L)				
n_(available)_	12	16	4	0.39
Mean (± SD)	8.5 (± 10.6)	5.2 (± 4.4)	2.0 (± 0.6)	
Median (range)	2.5 (0.9–31.0)	3.9 (1.4–18.4)	1.8 (1.6–2.9)	
> 1.69 mmol/L, n/n_(available)_ (%)	7/12 (58)	13/16 (81)	2/4 (50)	0.28
> 2.26 mmol/L, n/n_(available)_ (%)	6/12 (50)	12/16 (75)	1/4 (25)	0.14
> 5.65 mmol/L, n/n_(available)_ (%)	4/12 (33)	5/16 (31)	0/4 (0)	0.65
ALT (IU/L)				
n_(available)_	12	14	8	0.09
Mean (± SD)	48.0 (± 53.0)	89.0 (± 69.0)	54.0 (± 19.0)	
Median (range)	34.0 (14.0–198.0)	57.0 (20.0–232.0)	59.0 (30.0–78.0)	
> 35 IU/L, n/n_(available)_ (%)	6/12 (50)	10/14 (71)	5/8 (62)	0.61
> 55 IU/L, n/n_(available)_ (%)	2/12 (17)	7/14 (50)	4/8 (50)	0.14
AST (IU/L)				
n_(available)_	10	15	8	0.02
Mean (± SD)	34.0 (± 20.0)	65.0 (± 41.0)	58.0 (± 14.0)	
Median (range)	28.0 (15.0–76.0)	49.0 (26.0–187.0)	60.0 (36.0–78.0)	
> 35 IU/L, n/n_(available)_ (%)	4/10 (40)	12/15 (80)	8/8 (100)	0.01
> 48 IU/L, n/n_(available)_ (%)	2/10 (20)	8/15 (53)	6/8 (75)	0.08
GGT (IU/L)				
n_(available)_	5	8	2	
Mean (± SD)	59 (± 66)	58 (± 26)	121 (0)	0.16
Median (range)	44 (13–175)	55 (31–96)	121 (121–121)	
Creatine kinase				
n_(available)_	5	7	8	0.002
Mean (± SD)	76 (± 53)	131 (± 78)	2291 (± 1716)	
Median (range)	63 (16–160)	82 (60–249)	1810 (101–6000)	

Data were obtained at the time of patient diagnosis and during leptin-replacement naïve follow-up visits as permitted by available medical records. Statistical comparisons were made using median values. ^*^P-value for comparison between CGL1 and CGL2 groups only. CGL4 group omitted due lack of data. *ALT* Alanine aminotransferase, *AST* Aspartate aminotransferase, *CGL* Congenital generalized lipodystrophy, *FPG* Fasting plasma glucose, *HbA1c* Glycated hemoglobin, *n* Number of patients exhibiting the disease characteristic, *n*_(available)_, number of patients with available data, *SD* Standard deviation

No statistical differences in median values for HbA1c, triglycerides, FPG, fasting insulin, GGT and ALT were observed between subtypes (*P* > 0.05). However, a significant difference in median values for AST was observed between the subtypes (*P* = 0.02) with the highest value observed in patients with CGL4 (60 IU/L) and the lowest value observed in those with CGL1 (28 IU/L). In addition, median creatine kinase values differed between subtypes (*P* = 0.002) with the highest value occurring in CGL4 (1810 IU/L) with lower values for CGL1 (63 IU/L) and CGL2 (82 IU/L). Differences were also observed between the median total cholesterol values (*P* = 0.04) with the highest value detected in CGL1 (5.2 mmol/L) and lower values in CGL2 (4.3 mmol/L) and CGL4 (3.6 mmol/L).

### Characteristics of patients diagnosed with CGL at or before 1 year of age

Twenty-two patients were diagnosed with CGL at or before the age of 1 year. Demographics, clinical characteristics, and metabolic parameters for this subgroup of patients detailed in Table 4. Forty-four percent (n = 8/18) of these patients had acromegaloid features and 33% (n = 6/18) had acanthosis nigricans. Of the 19 females in this subgroup, four (21%) had hirsutism. At least one organ system abnormality occurred in 77% (n = 17/22) of patients with the liver (68%, n = 15/22), cardiovascular system (41%, n = 9/22), and spleen (36%, n = 8/22) the most frequently affected organ systems. The only background treatments used in this subgroup were lipid-lowering treatments (omega-3 and low-fat formula) and cardiovascular medications (beta-blockers), each used in two patients (9%).

**Table 4:** Disease characteristics and metabolic parameters in patients diagnosed with CGL at or before 1 year of age

	Patients diagnosed ≤ 1 year of age
*Sex, n/n*_*(available)*_ *(%)*	
Female	19/22 (86)
Male	3/22 (14)
*Physical features, n/n*_*(available)*_ *(%)*	
Acanthosis nigricans	6/18 (33)
Acromegaloid features	8/18 (44)
Hirsutism*	4/19 (21)
*Organ system abnormalities, n/n*_*(available)*_ *(%)*	
Bones/joints	1/22 (5)
Cardiovascular system	9/22 (41)
Liver	15/22 (68)
Hepatomegaly	15/22 (68)
Pancreas	1/22 (5)
Renal system	4/22 (18)
Spleen	8/22 (36)
Splenomegaly	8/22 (36)
*Medication history, n/n*_*(available)*_ *(%)*	
Anticoagulants	0/22 (0)
Antidiabetic medication	0/22 (0)
Cardiovascular medication	2/22 (9)
Lipid-lowering medication/treatment	2/22 (9)
*Metabolic parameters*	
FPG (mmol/L)	
n_(available)_	11
Mean (± SD)	7.0 (± 3.5)
Median (range)	5.4 (4.0–14.0)
HbA1c (%)	
n_(available)_	13
Mean (± SD)	6.1 (± 1.9)
Median (range)	5.6 (4.5–11.5)
> 5.7%, n/n_(available)_ (%)	5/13 (38)
> 6.5%, n/n_(available)_ (%)	3/13 (23)
> 8.0%, n/n_(available)_ (%)	2/13 (15)
Total cholesterol (mmol/L)	
n_(available)_	14
Mean (± SD)	4.6 (± 1.4)
Median (range)	(2.1–7.7)
> 5.18 mmol/L, n/n_(available)_ (%)	4/14 (29%)
Triglycerides (mmol/L)	
n_(available)_	17
Mean (± SD)	4.1 (± 4.5)
Median (range)	2.4 (0.9–18.4)
> 1.69 mmol/L, n/n_(available)_ (%)	10/17 (59)
> 2.26 mmol/L, n/n_(available)_ (%)	9/17 (53)
> 5.65 mmol/L, n/n_(available)_ (%)	4/17 (24)
ALT (IU/L)	
n_(available)_	16
Mean (± SD)	70 (± 51)
Median (range)	49 (28–173)
> 35 IU/L, n/n_(available)_ (%)	12/16 (75)
> 55 IU/L, n/n_(available)_ (%)	7/16 (44)
AST (IU/L)	
n_(available)_	18
Mean (± SD)	63 (± 36)
Median (range)	54 (29–187)
> 35 IU/L, n/n_(available)_ (%)	16/18 (89)
> 48 IU/L, n/n_(available)_ (%)	10/18 (56)
GGT (IU/L)	
n_(available)_	10
Mean (± SD)	77 (± 52)
Median (range)	66 (18–175)
Creatine kinase (IU/L)	
n_(available)_	11
Mean (± SD)	1256 (± 1793)
Median (range)	160 (60–6000)

^*^Female patients only

Data were obtained at the time of patient diagnosis and during leptin-replacement naïve follow-up visits as permitted by available medical records. *ALT* Alanine aminotransferase, *AST* Aspartate aminotransferase, *CGL* Congenital generalized lipodystrophy, *FPG* Fasting plasma glucose, *GGT* Gamma-glutamyl transferase, *HbA1c* Glycated hemoglobin, *n* Number of patients exhibiting the disease characteristic, *n*_(available)_ Number of patients with available data, *SD* Standard deviation

Analysis of metabolic parameters for this subgroup revealed a mean HbA1c of 6.1% (± 1.9%), a mean FPG of 7.0 mmol/L (± 3.5 mmol/L) and mean triglycerides of 4.1 mmol/L (± 4.5 mmol/L). Thirty-eight percent (n = 5/13) and 53% (n = 9/17) of patients had HbA1c > 5.7% and triglycerides > 2.26 mmol/L, while 44% (n = 7/16) of patients had ALT > 55 IU/L and 56% (n = 10/18) had AST > 48 IU/L.

### Assessment of clinical characteristics and metabolic abnormalities between age groups

To explore the potential effect of age on clinical characteristics in CGL, we compared the prevalence of organ abnormalities and metabolic parameters between patients diagnosed in childhood (< 12 years of age) and patients diagnosed in adolescence or later (≥ 12 years of age). Demographic data for these subgroups are provided in Additional file 1: Table 4. Notably, the proportions of patients with abnormalities in the pancreas (38% versus 3%, *P* = 0.02) and bones and/or joints (62% versus 9%, *P* = 0.003) were higher in the older subgroup. Abnormalities affecting the spleen occurred in 40% of patients in the younger subgroup but were not reported in the older subgroup (*P* = 0.04) (Table 5).

**Table 5:** Disease characteristics and metabolic parameters for patients diagnosed < 12 years of age versus patients diagnosed ≥ 12 years of age

Characteristic	Patients diagnosed < 12 years of age	Patients diagnosed ≥ 12 years of age	*P*-value
*Age at diagnosis, years*			
n_(available)_	35	8	
Mean (± SD)	1.5 (± 2.3)	20.9 (± 10.0)	
Median (range)	1 (at birth–11)	17 (12–37)	
*Sex, n/n*_*(available)*_ *(%)*	35	8	
Female	30 (86)	7 (87)	
Male	5 (14)	1 (13)	
*Physical features, n/n*_*(available)*_ *(%)*			
Acanthosis nigricans	14/31 (45)	6/8 (75)	0.24
Acromegaloid features	20/31 (65)	5/8 (62)	1.00
*Organ system abnormalities, n/n*_*(available)*_ *(%)*			
At least one abnormality	27/35 (77)	8/8 (100)	0.32
Bones and/or joints	3/35 (9)	5/8 (62)	0.003
Cardiovascular system	15/35 (43)	1/8 (13)	0.22
Liver	23/35 (66)	7/8 (87)	0.40
Hepatomegaly	22/35 (63)	5/8 (62)	1.00
Pancreas	1/34 (3)	3/8 (38)	0.02
Renal system	6/35 (17)	4/8 (50)	0.07
Spleen	14/35 (40)	0/8 (0)	0.04
Splenomegaly	14/35 (40)	0/8 (0)	0.04
*Medication history, n/n*_*(available*)_ *(%)*			
Anticoagulants	0/35 (0)	1/8 (13)	0.19
Antidiabetic medication	2/35 (6)	7/8 (87)	< 0.001
Cardiovascular medication	5/35 (14)	2/8 (25)	0.60
Lipid-lowering medication/treatment	2/35 (11)	6/8 (75)	0.001
*Metabolic parameters*			
FPG (mmol/L)			
n_(available)_	17	6	0.01
Mean (± SD)	6.7 (± 3.3)	15.2 (± 5.8)	
Median (range)	5.0 (4.0–14.0)	17.9 (4.6–19.3)	
HbA1c (%)			
n_(available)_	21	7	< 0.001
Mean (± SD)	6.0 (± 1.9)	11.2 (± 3.1)	
Median (range)	5.4 (4.5–11.5)	12.0 (5.7–14.3)	
> 5.7%, n/n_(available)_	7/21 (33)	6/7 (86)	0.03
> 6.5%, n/n_(available)_ (%)	5/21 (24)	6/7 (86)	0.007
> 8.0%, n/n_(available)_ (%)	3/21 (14)	6/7 (86)	0.001
Total cholesterol (mmol/L)			
n_(available)_	23	6	0.02
Mean (± SD)	4.4 (± 1.1)	7.1 (± 3.2)	
Median (range)	4.1 (2.1– 7.7)	6.6 (3.9–13.2)	
Triglycerides (mmol/L)			
n_(available)_	26	7	0.003
Mean (± SD)	3.9 (± 3.9)	14.5 (± 10.7)	
Median (range)	2.4 (0.9–18.4)	12.4 (2.1–31.0)	
> 1.69 mmol/L, n/n_(available)_ (%)	16/26 (62)	7/7 (100)	0.07
> 2.26 mmol/L, n/n_(available)_ (%)	14/26 (54)	6/7 (86)	0.20
> 5.65 mmol/L, n/n_(available)_ (%)	5/26 (19)	5/7 (71)	0.02
ALT (IU/L)			
n_(available)_	28	7	0.29
Mean (± SD)	68.0 (± 55.0)	56.0 (± 64.0)	
Median (range)	48.0 (14.0–232.0)	45.0 (14.0–198.0)	
> 35 IU/L, n/n_(available)_ (%)	18/28 (64)	4/7 (57)	1.00
> 55 IU/L, n/n_(available)_ (%)	12/28 (43)	1/7 (14)	0.22
AST (IU/L)			
n_(available)_	28	6	0.01
Mean (± SD)	58.0 (± 33.0)	30.0 (± 19.0)	
Median (range)	51.0 (19.0–187.0)	22.0 (15.0–63.0)	
> 35 IU/L, n/n_(available)_ (%)	23/28 (82)	2/6 (33)	0.03
> 48 IU/L, n/n_(available)_ (%)	15/28 (54)	1/6 (17)	0.18
GGT (IU/L)			
n_(available)_	14	2	0.87
Mean (± SD)	69.0 (± 47.0)	47.0 (± 4.0)	
Median (range)	59.0 (13.0–175.0)	47.0 (44.0–50.0)	
Creatine kinase (IU/L)			
n_(available)_	17	3	0.02
Mean (± SD)	1145 (± 1591)	51 (± 31)	
Median (range)	229 (60–6000)	61 (16–76)	

Data were obtained at the time of patient diagnosis and during leptin-replacement naïve follow-up visits as permitted by available medical records. Statistical comparisons were made using median values. *ALT* Alanine aminotransferase, *AST* Aspartate aminotransferase, *CGL* Congenital generalized lipodystrophy, *FPG* Fasting plasma glucose, *GGT* Gamma-glutamyl transferase, *HbA1c* Glycated hemoglobin, *n* Number of patients exhibiting the disease characteristic, *n*_(available)_ Number of patients with available data; SD, standard deviation

Statistical analysis revealed higher median values in the older subgroup for HbA1c (12.0 versus 5.4%, *P* < 0.001), triglycerides (12.4 versus 2.4 mmol/L, *P* = 0.003), FPG (17.9 versus 5.0 mmol/L, *P* = 0.01), and total cholesterol (6.6 versus 4.1 mmol/L, *P* = 0.02). In contrast, median AST values (51 versus 22 IU/L, *P* = 0.01) and creatine kinase values (229 versus 61 IU/L, *P* = 0.02) were higher for the younger subgroup. No differences were observed between the subgroups for ALT or GGT (Table 5).

## Discussion

We present analysis of the largest CGL dataset collected from patients across the MENA region. This research supplements earlier CGL case studies from the region and supports the early onset and progression of comorbidities in the absence of lipodystrophy-specific treatment.

Previous work has shown that the CGL1 and CGL2 subtypes account for approximately 90% of CGL cases with a known genetic etiology, while CGL3 and CGL4 are extremely rare with only a small number of cases reported to date [11, 17, 26]. As with previous studies, CGL1 and CGL2 were the predominant subtypes in the MENA cohort, accounting for 76% of patients, with the remaining 24% of patients diagnosed with CGL4 [11, 17, 22, 26].

CGL exhibits autosomal recessive inheritance and therefore would be expected to affect male and females equally; however, there was a notable underrepresentation of male patients in the MENA cohort (14%). Previous studies of generalized lipodystrophy have shown a greater proportion of female patients ranging between 55.0% and 77.3% [2, 11, 17, 22, 26, 33–35]. Although the reasons for the marked underrepresentation of male patients in the MENA cohort are unclear, it is possible that loss of subcutaneous fat loss is less recognizable in young males; for example, it has been reported that low body fat in young males can overlap with normal variation [1, 12, 36]. Reproductive abnormalities (e.g. PCOS, oligoamenorrhea and hirsutism) can also affect females with lipodystrophy and may help diagnosis [10, 37, 38]. In support of this, seven female patients from the MENA cohort (all diagnosed with CGL before or at the age of 2 years) displayed hirsutism.

A key finding of our analysis was the young age at which patients in the MENA CGL were diagnosed. The mean age of diagnosis in the overall cohort was 5.1 years with 51% of patients diagnosed ≤ 1 year old and 81% diagnosed aged < 12 years. This mean age of diagnosis is markedly lower than the 12.3 (± 13.7) years reported for an international cohort of patients with generalized lipodystrophy from Brazil, Turkey, and the USA (89% of patients with CGL) [11]. However, the age range of CGL diagnosis for the MENA cohort (at birth to 37 years) is consistent with that reported in this international cohort as well as generalized lipodystrophy cohorts from Spain and Turkey [11, 17, 34, 35].

Generalized fat loss in CGL typically occurs at birth or soon thereafter, leading to diagnosis in infancy for some cases as supported here and by earlier work [2, 17, 26, 34]. However, some patients do not receive a definitive diagnosis of CGL until puberty or adulthood by which stage severe metabolic disease may have developed [11, 17, 26, 39–41]. In the MENA cohort, 19% of patients were diagnosed ≥ 12 years of age. It is possible that unfamiliarity with lipodystrophy syndromes (due to their rarity) among the wider medical community may account, in part, for delayed diagnosis of these patients as documented previously [1, 12, 14].

Parental consanguinity and family history are common findings in CGL [2, 4]. In the MENA cohort, the prevalence of family history of lipodystrophy (67%) and parental consanguinity (93%) was high. This rate of parental consanguinity is broadly comparable to that reported in CGL cohorts from Turkey (82%, based on family-level analysis) and Brazil (72%) but is higher than that reported in a systematic review of global CGL cases (61%) [2, 17, 22]. Several studies have highlighted the increased rate of consanguineous marriage in the MENA region (estimated between 20 and 50%) compared with the global estimate of 10% [42–45]. Consanguineous marriage increases the risk of an inheriting recessive disease mutations from a common ancestor and is a likely contributor to the etiology of CGL in the MENA region and elsewhere [2, 7, 17, 22, 43, 46]. Our data also suggest that parental consanguinity and family history may support the early identification of CGL patients in the MENA region through family screening [47].

International and regional natural history studies of generalized lipodystrophy have provided a wide range of prevalence estimates for physical characteristics and organ system abnormalities in the absence of lipodystrophy-specific treatment. These include acromegaloid features (up to 96% of patients), acanthosis nigricans (up to 89% of patients), as well as estimates for abnormalities of the liver (up to 89% of patients), spleen (up to 77% of patients), renal system (up to 54% of patients), cardiovascular system (up to 46% of patients) and pancreatitis (up to 31%) [2, 11, 22, 26, 35]. In the overall MENA cohort, at least one organ system abnormality was detected in 81% of patients, while the proportions of patients with individual organ system abnormalities (e.g., liver, cardiovascular system, renal system) are consistent with those reported in earlier studies [2, 11, 22, 26, 35]. Possible reasons for the broad variation in organ system prevalence estimates observed among natural history studies of generalized lipodystrophy include differences in the ages and genetic backgrounds of the patients examined, and differences in the diagnosis and reporting of cases between geographical regions.

Severe metabolic complications were also observed in the MENA cohort. For example, the proportion of patients with HbA1c > 6.5% (39%) and triglycerides > 5.65 mmol/L (30%) was comparable to that reported for the international generalized lipodystrophy cohort (43% and 51%, respectively) [11]. Also, the mean HbA1c (7.3%), median triglycerides (2.9 mmol) and median FPG (5.4 mmol) for the overall MENA cohort were comparable to corresponding values reported for a Brazilian CGL cohort (mean HbA1c: 7.4%; median triglycerides: 3.1 mmol/L [= 276 mg/dl]; median FPG: 4.9 mmol/L [= 89 mg/dl]) [22].

Notably, our analysis revealed that the disease burden of CGL manifests at a young age. Of the patients who were diagnosed at or before 1 year of age, 44% and 33% presented with acromegaloid features and acanthosis nigricans, respectively, 77% had at least one organ abnormality, 23% had HbA1c > 6.5% and 24% had triglycerides > 5.65 mmol/L. The development of the organ system and metabolic complications during early childhood has been documented in several CGL cases studies [23, 48–51]. For example, Eltermann et al. (2010) reported severe metabolic complications including elevated AST (104 IU/L), ALT (203 IU/L), GGT (508 IU/L), and triglycerides of 23.7 mmol/L (= 2100 mg/dL) in a 3-week-old patient with CGL2 of Turkish origin [51]. Additionally, a CGL case series involving a subgroup of seven patients from Oman aged between 0 and 4 years, reported that all patients had hepatomegaly, with splenomegaly (67%), acanthosis nigricans (67%), acromegaloid features (67%) and hypertriglyceridemia (71%) also detected [23]. Results from the MENA cohort and other studies therefore suggest that metabolic and organ system abnormalities in CGL can develop around the time of onset of fat loss and may be detectable during infancy [23, 48–51].

Although most patients in the MENA dataset were diagnosed with CGL during childhood (< 12 years of age, 81%), an appreciable proportion (19%) were diagnosed in adolescence or later (≥ 12 years of age). Comparison of the metabolic parameters between these two subgroups enabled exploratory analysis of the effect of age on the progression of metabolic disease. Median HbA1c, FPG, triglycerides and total cholesterol levels were statistically higher in patients diagnosed ≥ 12 years of age versus those diagnosed < 12 years of age. These findings suggest that in the absence of lipodystrophy-specific therapies, CGL complications worsen with age. This observation is supported by previous work whereby the severe metabolic complications of generalized lipodystrophy (e.g., diabetes and hypertriglyceridemia) were shown to develop around adolescence and progressed further during adulthood [11, 26]. Similarly, a study conducted at the US National Institute of Health, which included patients with both generalized and partial forms of lipodystrophy, showed that at baseline, adolescent patients (aged > 12 and < 18 years) had a greater severity of metabolic disease versus children (aged < 12 years) [52].

The phenotypic characteristics associated with lipodystrophy largely overlapped between the CGL subtypes in the MENA cohort. Notable exceptions were acanthosis nigricans (greater prevalence in CGL1 and CGL2 versus CGL4), cardiovascular abnormalities (greater prevalence in CGL2 and CGL4 versus CGL1), AST levels (highest median value for CGL4) and creatine kinase levels (highest in CGL4). Although these exceptions may be due to differences in the age of diagnosis between the CGL subtypes (patients with CGL1 were, on average, diagnosed later than patients with CGL2 and CGL4), they may also reflect, in part, the distinct clinical features of the different subtypes [17, 26].

The MENA cohort included 10 patients who were diagnosed with CGL4, a rare lipodystrophy subtype caused by variants in the *CAVIN1* gene that was first documented in 2002 [53–55]. Since then, approximately 30 cases have been reported in the literature [55]. *CAVIN1* encodes the 390-amino acid cavin-1 protein, which is involved in the biogenesis of caveolae, small (50–100 nm) bulb-shaped folds found on the plasma membrane of adipocytes, endothelial cells, myocytes, and fibroblasts. These structures play a role in intracellular trafficking and signaling, and the homeostasis of cholesterol, fatty acids, and triglycerides [56, 57]. Patients with CGL4 often have some body fat at birth which is gradually lost during infancy; however, mechanical and bone marrow fat is typically preserved [13]. Clinical characteristics unique to CGL4 include myopathy, local percussion-induced muscle ‘mounding’, pyloric stenosis, osteopenia, and joint contractures [13]. Patients with CGL4 may also have a predisposition to cardiac events (e.g., cardiac arrhythmias, and prolonged QT intervals and exercise-induced ventricular tachycardia) and display milder metabolic disease compared with other CGL subtypes [13, 16].

In general, the clinical and metabolic characteristics of MENA patients with CGL4 were comparable to those for the other CGL subtypes. However, the prevalence of cardiovascular abnormalities in MENA patients with CGL4 (60%) lends some support for the development of increased cardiac risk at an early age in this lipodystrophy subtype. It is also possible that the elevated AST levels in the MENA CGL4 subtype may be due to increased muscle abnormalities as supported by the markedly higher creatine kinase levels compared with CGL1 and CGL2 [16, 58, 59]. Of note, all CGL4 patients in the MENA cohort were diagnosed before the age of 12 years and these patients contributed to the higher creatine kinase levels observed for this subgroup versus patients from the MENA cohort diagnosed ≥ 12 years.

This research has several strengths. It adds to the growing body of evidence describing the natural history of this CGL and provides new information on CGL cases from a geographical region that hitherto had limited data. Our dataset also comprised a heterogeneous patient population who received a diagnosis ranging from the time of birth to adulthood. As such, this enabled investigation of disease characteristics during infancy, and an exploration of the effect of age on CGL progression.

Our analysis has a number of limitations. Firstly, the data were collected from historical medical records at the time of patient diagnosis and during leptin-replacement naïve follow-up visits, some of which had incomplete entries. This limited the amount of available data for analysis for some variables. Secondly, some variables, particularly those related to physical characteristics, organ abnormalities, and background medication, were largely identified as being either ‘present’ or ‘absent’ based on available data recorded in patient medical records. Consequently, we provide a simplified analysis of the onset and progression of CGL organ abnormalities. Furthermore, data were not standardized prior to the start of this research, while variations in data collection, clinical protocols, documentation procedures, and measurement methodologies between the participating centers could impact the availability of data for the variables analyzed. Thirdly, 77% of patients in the overall cohort were collected from Saudi Arabia and Oman, which may limit the generalizability of our findings across the MENA region and other regions. Fourthly, there was a lack of granular detail in the genetic testing for a small number of patients in our cohort. Finally, 86% of patients in the MENA cohort were female leading to a paucity of data for male patients with CGL in the region.

## Conclusion

This analysis of the largest CGL cohort from the MENA region provides new information regarding the diagnosis and burden of this disease. Our findings support previous studies which show that organ system damage and metabolic complications in CGL can occur early in life and progress with age. Future investigations of CGL cases from the MENA region, including those receiving lipodystrophy-specific therapies, have the potential to improve treatment strategies for patients with CGL in this region and elsewhere.

### Supplementary Information

**Additional file 1.** Genetic testing and DNA sequence analysis.

## Data Availability

The data collected have been reported in the results. For further information, please contact author for specific data requests.

## Abbreviations

AGL: Acquired generalized lipodystrophy
*AGPAT2*: 1-Acylglycerol-3-phosphate O-acyltransferase 2
ALT: Alanine aminotransferase
APL: Acquired partial lipodystrophy
AST: Aspartate aminotransferase
*BSCL2*: Berardinelli-Seip congenital lipodystrophy 2
*CAV1*: Caveolin 1
*CAVIN1*: Caveolae-associated protein 1
CGL: Congenital generalized lipodystrophy
CGL1: Congenital generalized lipodystrophy type 1
CGL2: Congenital generalized lipodystrophy type 2
CGL3: Congenital generalized lipodystrophy type 3
CGL4: Congenital generalized lipodystrophy type 4
FPG: Fasting plasma glucose
GGT: Gamma-glutamyl transferase
HbA1c: Glycated hemoglobin
MENA: Middle East and North Africa
NAFLD: Non-alcoholic fatty liver disease
*PTRF*: Polymerase I and transcript release factor
UAE: United Arab Emirates
