# Supplementary material for: Analysis of disease characteristics of a large patient cohort with congenital generalized lipodystrophy from the Middle East and North Africa
Source: Orphanet J Rare Dis. 2024 Mar 13;19:118. doi: 10.1186/s13023-024-03084-2 (PMC10935864; doi:10.1186/s13023-024-03084-2)
Supplement: Supplementary file 1 — Additional file 1. Genetic testing and DNA sequence analysis. [file 13023_2024_3084_MOESM1_ESM.docx]

**Analysis of disease characteristics of a large patient cohort with congenital generalized lipodystrophy from the Middle East and North Africa**

Saif Al Yaarubi et al. (OJRD-D-23-00237R1)

**Supplementary material**

**Genetic testing and DNA sequence analysis**

Genetic analyses were conducted at accredited diagnostic laboratories using their in-house, proprietary sequencing techniques or were performed at participating medical centers using previously published methods [1-3]. For samples analyzed at accredited diagnostic laboratories, DNA sequencing technologies included exome sequence capture performed via genomic hybridization techniques (e.g., Agilent SureSelect technology, Agilent Technologies, USA; Roche NimbleGen technology, Roche, USA) followed by high-throughput sequencing. Sequence reads were aligned to the human reference genome at the time of analysis using their proprietary bioinformatic analysis pipelines. The clinical relevance of variants related to CGL-associated genes (*AGPAT2*, *BSCL2*, *CAV1*, *CAVIN1*) was assessed according to the criteria established by the American College of Medical Genetics [4]. Assessment of the effect of non-synonymous DNA sequence variants effect used multiple algorithms including MutationTaster with PolyPhen-2 [5], SIFT [6], and MutationTaster2 [7]. The Reference SNP cluster ID (rsID) or variant description based on the nomenclature of the Human Genome Variation Society (HGSV; https://hgvs-nomenclature.org/stable/) for each variant was identified using the VarSome human genomic variant search engine [8].

The location and the name of the accredited diagnostic laboratories used in this research were:

- Germany
  - Bioscientia Institut für Medizinische Diagnostik GmbH Humangenetik, Ingelheim (<https://genetik.bioscientia.de/>)
  - Centogene AG, Am Strande 7, Rostock, Mecklenburg-Vorpommern (<https://www.centogene.com/)>
- India
  - MedGenome Labs Ltd. 3rd Floor, Narayana Nethralaya Building, Narayana Health City, Bangalore (<https://diagnostics.medgenome.com/>)
- Republic of Korea
  - 3billion, Inc.14th, 416 Teheran-ro, Gangnam-gu, Seoul (<https://3billion.io/index>)
- Saudi Arabia
  - Molecular Diagnostic Laboratory, King Faisal Specialist Hospital Research Centre, Riyadh

(<https://www.kfshrc.edu.sa/en/home/hospitals/riyadh/medicalgenetics>)

- United States of America
  - Medical Neurogenetics, LLC, 5424 Glenridge Dr, Atlanta, Georgia, (<https://mnglabs.labcorp.com/>)
  - Fulgent Genetics, 4399 Santa Anita Ave. El Monte, California (<https://www.fulgentgenetics.com/>)
  - Precision Genetics, 430 Roper Mountain Road, Suite B, Greenville, South Carolina (<https://precisiongenetics.com>)

**Supplementary Table 1.** Genotypic information

| **CGL classification / variant information** | **rsID/HGVS** | **n**  **(total = 42)*** | **Clinical relevance** |
| --- | --- | --- | --- |
| **CGL1** |  | **14** |  |
| *AGPAT2*: c.335del (p.Pro112Argfs*39)^†,9^ | rs1588263957 | 8 | Pathogenic |
| *AGPAT2*: c.158del (p.Gly53Alafs*8) ^†,10^ | rs2131023674 | 4 | Pathogenic |
| Variant not determined |  | 2 |  |
| **CGL2** |  | **18** |  |
| *BSCL2*: c.750dup (p.Leu251Alafs*23)^11^ | rs1554983076 | 3 | Pathogenic |
| *BSCL2*: c.502C>T (p.Gln168Ter) ^†,12^ | rs1945346386 | 1 | Pathogenic |
| *BSCL2*: c.942dup (p.Leu315fs*23) ^†,13^ | *BSCL2*(NM_032667.6):c.942dup | 5 | Pathogenic |
| *BSCL2*: c.672-2A>C^†,14^ | rs766061024 | 4 | Pathogenic |
| *BSCL2*: c.404G>C (p.Arg135Thr) ^†,15^ | *BSCL2*(NM_032667.6):c.404G>C | 1 | Variant of uncertain significance |
| *BSCL2*: c.412C>T^†,16^ | rs137852970 | 1 | Pathogenic |
| *BSCL2*: c.573 +1 C>T | N/A | 1 | Pathogenic |
| Variant not determined |  | 2 |  |
| **CGL4** |  | **10** |  |
| *CAVIN1/PTRF*: c.160del (p.Val54Cysfs*2)^†,17^ | rs1567782465 | 9 | Pathogenic |
| *CAVIN1/PTRF*: c.638T>C (p.Ile213Thr) | rs146929422 | 1 | variant of uncertain significance |

*One patient with CGL in the overall cohort of 43 patients had an unknown genetic profile and was not classified into a CGL subgroup..

^†^ Reported in previous studies and/or analysis of CGL

HGVS, Human Genome Variation Society; rsID, Reference SNP cluster ID

**Supplementary Table 2.** The proportion of patients in the overall MENA cohort with elevated metabolic values

|  | n_(available)_ | n (%) |
| --- | --- | --- |
| ALT > 35 U/L | 35 | 22 (63) |
| ALT > 55 U/L | 35 | 13 (37) |
| AST > 35 U/L | 34 | 25 (74) |
| AST > 48 U/L | 34 | 16 (47) |
| HbA1c > 5.7% | 28 | 13 (46) |
| HbA1c > 6.5% | 28 | 11 (39) |
| HbA1c > 8.0% | 28 | 9 (32) |
| Total cholesterol > 5.18 mmol/L | 29 | 9 (31) |
| Triglycerides > 1.69 mmol/L | 33 | 23 (70) |
| Triglycerides > 2.26 mmol/L | 33 | 20 (61) |
| Triglycerides > 5.65 mmol/L | 33 | 10 (30) |

Data were obtained at the time of patient diagnosis and during leptin replacement naïve follow-up visits as permitted by available medical records. Metabolic thresholds were taken from previously published material [please refer to main manuscript]. ALT, alanine aminotransferase; AST, aspartate aminotransferase; CGL, congenital generalized lipodystrophy; FPG, fasting plasma glucose; GGT, gamma-glutamyl transferase; HbA1c, glycated hemoglobin; n, number of patients exhibiting the disease characteristic; n_(available)_, number of patients with available data; SD, standard deviation.

**Supplementary Table 3.** Demographics stratified by CGL subtype

|  | **CGL1 subtype** | **CGL2 subtype** | **CGL4 subtype** |
| --- | --- | --- | --- |
| Age at diagnosis, years | | | |
| n_(available)_ | 14 | 18 | 10 |
| Mean (±SD) | 9.7 (±10.6) | 3.3 (±8.4) | 0.8 (±0.8) |
| Median (range) | 7.0 (0.1–37.0) | 0.8 (0.1–36.0) | 0.5 (at birth–2.0) |
| Sex, n/n_(available)_ (%) | | | |
| Female | 13/14 (93) | 16/18 (89) | 7/10 (70) |
| Male | 1/14 (7) | 2/18 (11) | 3/10 (30) |
| Country, n/n_(available)_ (%) | | | |
| Egypt | 0/14 (0) | 1/18 (6) | 0/10 (0) |
| Iraq | 0/14 (0) | 1/18 (6) | 0/10 (0) |
| Kingdom of Bahrain | 0/14 (0) | 1/18 (6) | 0/10 (0) |
| Libya | 1/14 (7) | 2/18 (11) | 0/10 (0) |
| Oman | 0/14 (0) | 4/18 (22) | 9/10 (90) |
| Palestine* | 0/14 (0) | 1/18 (6) | 0/10 (0) |
| Saudi Arabia | 12/14 (86) | 8/18 (44) | 0/10 (0) |
| UAE | 1/14 (7) | 0/18 (0) | 1/10 (10) |
| Consanguinity, n/n_(available)_ (%) | 14/14 (100) | 15/18 (83) | 10/10 (100) |
| Family history of lipodystrophy, n/n_(available)_ (%) | 9/14 (64) | 12/18 (67) | 8/10 (80) |
| Height (cm) | | | |
| n_(available)_ | 10 | 11 | 10 |
| Mean (±SD) | 111.0 (±46.0) | 91.0 (±36.0) | 67.0 (±15.0) |
| Median (range) | 91.0 (60.0–174.0) | 80.0 (60.0–168.0) | 70.0 (45.0–87.0) |
| Weight (kg) | | | |
| n_(available)_ | 10 | 12 | 10 |
| Mean (±SD) | 28.0 (± 23.9) | 16.7 (±19.2) | 6.8 (±3.2) |
| Median (range) | 13.5 (5.3–68.0) | 9.9 (3.0–68.0) | 7.8 (2.4–10.3) |
| BMI (kg/m^2^) | | | |
| n_(available)_ | 10 | 9 | 10 |
| Mean (±SD) | 18.0 (±3.4) | 17.3 (±3.9) | 13.9 (±2.3) |
| Median (range) | 17.7 (13.0–25.0) | 18.0 (10.7–24.1) | 14.0 (9.9–16.9) |

*This patient was treated in a medical center in the UAE.

Data were obtained at the time of patient diagnosis and during leptin replacement naïve follow-up visits as permitted by available medical records. Proportions based on the number of patients with available data. BMI, body mass index; CGL, congenital generalized lipodystrophy; n, number of patients exhibiting the disease characteristic; n_(available)_, number of patients with available data; SD, standard deviation.

**Supplementary Table 4.** Demographics stratified by patients diagnosed < 12 years of age patients diagnosed ≥ 12 years of age

|  | **Patients diagnosed  < 12 years of age** | **Patients diagnosed  ≥ 12 years of age** |
| --- | --- | --- |
| Age at diagnosis, years | | |
| n_(available)_ | 35 | 8 |
| Mean (±SD) | 1.5 (±2.3) | 20.9 (±10.0) |
| Median (range) | 1.0 (at birth–11.0) | 17 (12–37.0) |
| Sex, n/n_(available)_ (%) | | |
| Female | 30/35 (86) | 7/8 (87) |
| Male | 5/35 (14) | 1/8 (13) |
| Height (cm) | | |
| n_(available)_ | 26 | 6 |
| Mean (±SD) | 76.0 (±20.0) | 164.0 (±6.0) |
| Median (range) | 75.0 (45.0–145.0) | 163.0 (155.0–174.0) |
| Weight (kg) | | |
| n_(available)_ | 27 | 6 |
| Mean (±SD) | 9.6 (±7.5) | 56.8 (±7.9) |
| Median (range) | 9.1 (2.4–40.0) | 56.5 (46.1–68.0) |
| BMI (kg/m^2^) | | |
| n_(available)_ | 24 | 6 |
| Mean (±SD) | 15.3 (±2.7) | 21.2 (±2.8) |
| Median (range) | 16.0 (9.9–19.2) | 20.6 (17.7–25.0) |

Data were obtained at the time of patient diagnosis and during leptin replacement naïve follow-up visits as permitted by available medical records. Proportions based on the number of patients with available data. BMI, body mass index; n, number of patients exhibiting the disease characteristic; n_(available)_, number of patients with available data; SD, standard deviation.

# **References**

1. Akinci B, Onay H, Demir T, Ozen S, Kayserili H, Akinci G, et al. Natural History of Congenital Generalized Lipodystrophy: A Nationwide Study From Turkey. *J Clin Endocrinol Metab*. 2016;**101**(7):2759-67.

2. Alfadhel M, Abadel B, Almaghthawi H, Umair M, Rahbeeni Z, Faqeih E, et al. HMG-CoA Lyase Deficiency: A Retrospective Study of 62 Saudi Patients. *Front Genet*. 2022;**13**:880464.

3. Alfares AA. Applying filtration steps to interpret the results of whole-exome sequencing in a consanguineous population to achieve a high detection rate. *Int J Health Sci (Qassim)*. 2018;**12**(5):35-43.

4. Richards S, Aziz N, Bale S, Bick D, Das S, Gastier-Foster J, et al. Standards and guidelines for the interpretation of sequence variants: a joint consensus recommendation of the American College of Medical Genetics and Genomics and the Association for Molecular Pathology. *Genet Med*. 2015;**17**(5):405-24.

5. Adzhubei IA, Schmidt S, Peshkin L, Ramensky VE, Gerasimova A, Bork P, et al. A method and server for predicting damaging missense mutations. *Nat Methods*. 2010;**7**(4):248-9.

6. Kumar P, Henikoff S, Ng PC. Predicting the effects of coding non-synonymous variants on protein function using the SIFT algorithm. *Nat Protoc*. 2009;**4**(7):1073-81.

7. Schwarz JM, Cooper DN, Schuelke M, Seelow D. MutationTaster2: mutation prediction for the deep-sequencing age. *Nat Methods*. 2014;**11**(4):361-2.

8. Kopanos C, Tsiolkas V, Kouris A, Chapple CE, Albarca Aguilera M, Meyer R, Massouras A. VarSome: the human genomic variant search engine. *Bioinformatics*. 2019;**35**(11):1978-80.

9. Hummadi A, Ali Nahari A, Jaber Alhagawy A, Zakri I, Abutaleb R, Yafei S. Congenital generalized lipodystrophy in two siblings from Saudi Arabia: A case report. Clin Case Rep. 2022 Apr; 10(4): e05720.

10. Abuzenadah A, Alganmi N, AlQurashi R, Hawsa E, AlOtibi A, Hummadi A et al. Familial Screening for the Prevention of Rare Diseases: A Focus on Lipodystrophy in Southern Saudi Arabia. J Epidemiol Glob Health. 2024 Jan 17. doi: 10.1007/s44197-023-00182-5.

11. https://www.ncbi.nlm.nih.gov/snp/rs1554983076#clinical_significance (last accessed, Feb 2024)

12. https://www.ncbi.nlm.nih.gov/snp/rs1945346386#variant_details (last accessed, Feb 2024)

13. https://www.ncbi.nlm.nih.gov/clinvar/variation/434545/(last accessed, Feb 2024)

1.4. https://www.ncbi.nlm.nih.gov/snp/rs766061024/#clinical_significance (last accessed, Feb 2024)

15. https://www.ncbi.nlm.nih.gov/clinvar/variation/871579/ (last accessed, Feb 2024)

16. https://www.ncbi.nlm.nih.gov/snp/rs137852970#clinical_significance (last accessed, Feb 2024)

17. https://www.ncbi.nlm.nih.gov/snp/rs1567782465#clinical_significance (last accessed, Feb 2024)
